# Frequency Bandwidth of Pressure Sensors Dedicated to Blast Experiments

**DOI:** 10.3390/s22103790

**Published:** 2022-05-17

**Authors:** Mathieu Chalnot, Patrick Pons, Hervé Aubert

**Affiliations:** Laboratoire d’Analyse et d’Architecture des Systèmes (LAAS-CNRS), Université de Toulouse, Centre National de la Recherche Scientifique (CNRS), Institut National Polytechnique de Toulouse (INPT), 7 Avenue du Colonel Roche, 31031 Toulouse, France; ppons@laas.fr (P.P.); haubert@laas.fr (H.A.)

**Keywords:** frequency bandwidth, pressure sensor, blast wave, Kingery and Bulmash data

## Abstract

New broadband (>1 MHz) pressure sensors are regularly reported in the literature to measure the overpressure of blast waves. However, the frequency bandwidth needed to accurately measure such overpressure has not yet been clearly discussed. In this article, we present a methodology to determine the bandwidth required to estimate the overpressure magnitude at the front of a blast wave, in order to obtain a desired estimation accuracy. The bandwidth is derived here by using Kingery and Bulmash data.

## 1. Introduction

During an explosion, the measurement of the overpressure at the front of the generated blast wave by using pressure sensors is a standard and well-controlled technique (see, e.g., [1,2,3,4,5,6,7,8]). Assessing the magnitude of the overpressure peak is crucial to characterize explosives, because this magnitude can be used to derive a plethora of physical quantities at the blast wave front from the Rankine–Hugoniot relationships [9]. Due to the ultra-fast variation in the overpressure generated during the explosion, the accurate measurement of the peak magnitude is very challenging in practice [10] and often requires broadband (>1 MHz) pressure sensors [1]. In order to overcome the frequency bandwidth limitations of commercially available pressure transducers, new sensing devices are regularly reported in the literature to measure the overpressure magnitude of blast waves (see, e.g., [11,12,13,14,15]). Without giving any justification for the bandwidth specification for air blast experiments, the authors have targeted the design of pressure sensors with a frequency bandwidth of, e.g., 100 kHz [14] or 1 GHz [11]. However, the sensor bandwidth needed to accurately estimate the magnitude of the overpressure peak has surprisingly not yet been derived. According to [16,17], the bandwidth requirement differs in small-scale and large scale experiments. In [15], the bandwidth is assumed to be 50 times larger than the attenuation rate of the overpressure peak, but no method is provided to obtain the attenuation rate. To date, the bandwidth requirement of pressure sensors has not been derived from physical considerations on the blast wave to be characterized, while this requirement may be very useful in selecting the appropriate sensor technology and minimize the fabrication cost. 

The frequency bandwidth of pressure sensors used to measure the magnitude of the overpressure peak at the front of a blast wave is the result of a trade-off. Indeed, if the bandwidth is oversized then the pressure signal may be significantly affected by thermal and/or shot noise (which degrades the estimation accuracy of the pressure), whereas an undersized bandwidth would not allow the accurate estimation of the overpressure peak magnitude due to the filtering of crucial high-frequency components of the signal. The objective of the present study is to determine the bandwidth of pressure sensors required to estimate the overpressure magnitude at the front of a blast wave, in order to achieve a desired estimation accuracy. We show that both the TriNitroToluene (TNT) equivalent of explosives and the so-called scaled distance play a crucial role in the determination of this frequency bandwidth.

The paper is organized as follows. In Section 2, we highlight and discuss the assumptions applied throughout the paper to determine the frequency bandwidth of sensors needed to estimate the overpressure magnitude at the front of a blast wave. Next, we predict in Section 3 the sensor bandwidth needed to ensure a desired estimation accuracy on the overpressure magnitude. The bandwidth is derived by using Kingery and Bulmash data. For the sake of clarity, illustrative examples related to surface and free-air bursts are analyzed. Conclusion and perspectives to this work are given in Section 4.

## 2. Materials and Methods

### 2.1. The Friedlander Waveform

**Figure 1:**
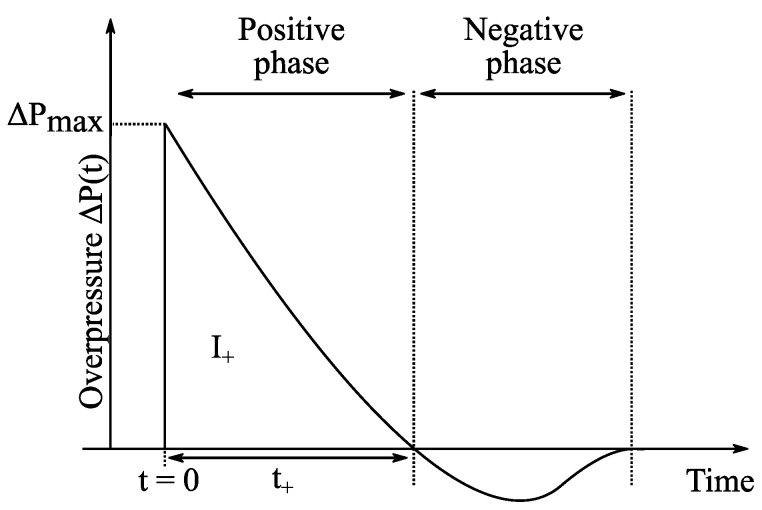
Typical variation in the overpressure at the front of a blast wave generated by the explosion of a load.

During the detonation of an explosive load, the abrupt release of energy occurs and a shockwave, namely a *blast wave*, is generated [18]. When the blast wave crosses a point in space at time t=0, the variation in the overpressure ΔP(t) at that point can be modeled by the so-called *modified Friedlander* waveform (or signal) given by (see, e.g., [19]):

(1a) $$\Delta P\left(t\right)=\Delta P_{MAX}\left(1-\frac{t}{t_{+}}\right)e^{-b\frac{t}{t_{+}}}\;\;\mathrm{for}\;t\geq 0$$

(1b) ΔP(t)=0  for t<0

where $\Delta P_{MAX}$, $t_{+}$ and b denote, respectively, the magnitude of the overpressure peak, the duration of the so-called *positive phase*, and the dimensionless decay coefficient of the pressure signal [20] (see Figure 1). According to [1], as soon as the peak is reached, the overpressure decays abruptly at a rate which depends on the *scaled distance* $Z=R/\sqrt[{3}]{m_{TNT}}$, where R is the separation distance (in meters) between the center of the explosive load and the pressure sensor, and $m_{TNT}$ is the equivalent mass of Trinitrotoluene (TNT) explosive (in kg) that would yield to the same blast wave at the same scaled distance Z. (Note that the Friedlander waveform of Equation (1) may be used to model the variation in many physical features of blast waves [19], and consequently the results reported in this paper are valid not only for the pressure measurement, but also for, e.g., gas density or velocity measurement).

Following the approximation reported in [1,7,19], we assume here that the pressure signal to be measured follows the Friedlander-type waveform of Equation (1), although the measured signals may eventually differ in practice from this simple model. Moreover, we assume that the magnitude of the overpressure peak is given by the highest value of the measured pressure signal obtained during the direct data acquisition. In other words, unlike the measurement technique applied in [7,19], the magnitude is not derived here from fitting the measured data with a Friedlander waveform.

In addition, we consider in this work the measurement by using sensors of both the side-on and reflected pressures. The reflected pressure is measured when the direction of the pressure flow is normal to the transducer surface, while the side-on pressure is measured when the flow direction is tangential to this surface.

Main descriptors of this waveform (or signal) are the magnitude of the overpressure peak ($\Delta P_{MAX}$), the positive pressure impulse ($I_{+}$), and the positive phase duration ($t_{+}$). The time of arrival of the blast wave will not be studied here and, consequently, the origin (t=0) indicates when the blast wave reaches the pressure sensor. Blast wave descriptors depend on the explosive type (e.g., C4, Semtex, TNT, etc.), the equivalent mass of TNT ($m_{TNT}$) of the explosive load and the scaled distance $Z=R/\sqrt[{3}]{m_{TNT}}$, where *R* is the separation distance between the center of the explosive load and the sensor.

### 2.2. The Kingery and Bulmash Data

We assume here that any explosive load is equivalent to a mass $m_{TNT}$ of TNT explosive. Based on the Kingery and Bulmash (K&B) data [2,21], blast wave descriptors can then be related to the scaled distance Z and equivalent mass $m_{TNT}$ of TNT explosive. In our work, we will use the simplified Kingery and Bulmash model reported in [22], where each of the measured descriptors $\Delta P_{MAX}$, $t_{+}$ and $I_{+}$ of blast waves are conveniently given for both side-on and reflected pressure measurement, and for hemispherical surface bursts and spherical airbursts. K&B data are a reference standard [7] and are used, for example, in the simulation software ConWep [23], in the LOAD_BLAST module of LS-DYNA [24] and in many research works (see, e.g., [3,4,6,20]).

Moreover, we assume that the Hopkinson scaling law is valid. This law states that during an explosion, the magnitude of the overpressure peak measured at the distance R from the equivalent mass $m_{TNT}$ of TNT is identical to the magnitude measured at the distance kR(for any k>1) from an equivalent mass $k^{3}m_{TNT}$ of TNT. Moreover, the positive phase duration $t_{+}$ and the positive phase impulse $I_{+}$ given by K&B data are linearly dependent on $\sqrt[{3}]{m_{TNT}}$ and, consequently, at a given scaled distance Z, they differ by a factor $\sqrt[{3}]{k}$ for two different explosive loads with equivalent masses of $m_{TNT}$ and $k\;m_{TNT}$ of TNT, respectively. According to [6], the accuracy of K&B data is questionable for scaled distances Z smaller than 2 m/kg^1/3^, and other reference data may be more accurate to estimate blast wave descriptors when, e.g., Semtex explosives are involved [8]. Keeping in mind these limitations, we derive from K&B data in the next sections the bandwidth of pressure sensors required to estimate the overpressure magnitude at the front of a blast wave, in order to achieve a desired estimation accuracy.

### 2.3. Definitions of the Cut-off Frequency, Resonant Frequency and Frequency Bandwidth of Pressure Sensors

A pressure sensor is usually modeled by a second-order low-pass filter, which exhibits a high cut-off frequency $f_{c}$ and a resonant frequency $f_{0}$. At the cut-off frequency, the power of the signal at the output of the sensor is half of the power delivered at its nominal operation. Because of its finite cut-off frequency $f_{c}$, the sensor cannot instantaneously provide the waveform applied at its input port. At the resonant frequency $f_{0}$, the power at the sensor output reaches its maximum value. This frequency is commonly specified in the sensor datasheet (see, e.g., [25]) and reported in publications on pressure sensors dedicated to blast wave experiments (see, e.g., [14]). When a blast wave impinges on the transducer surface, the resonant frequency is usually excited [26,27]. In order to mitigate its impact on the measured signal, a low-pass filtering is typically applied at the sensor output [28,29] and, consequently, the actual bandwidth of a pressure sensing system is lower than the resonant frequency (according to [30], the bandwidth is a fifth of the resonant frequency). 

For the sake of simplicity, the frequency response of a pressure sensor is modeled here by the transfer function H of a first-order low-pass filter, as follows:

(2) $$H\left(s\right)=\frac{v\left(s\right)}{p\left(s\right)}=k_{s}\frac{\omega_{c}}{s+\omega_{c}}$$

where v(s) is the Laplace transform of the voltage V(t) delivered at the sensor output, p(s) is the Laplace transform of the overpressure ΔP(t) applied to the surface of the pressure transducer (this overpressure is modeled here by the modified Friedlander waveform of Equation (1)), $k_{S}$ denotes the sensitivity of the transducer, $\omega_{c}=2\pi f_{c}$ is the cut-off angular frequency of the first-order low-pass filter model, and s is the s-plane variable of the Laplace transform. The frequency bandwidth of the sensor is then only limited by the cut-off frequency $f_{c}$.

## 3. Frequency Bandwidth of Pressure Sensors for Blast Experiments

### 3.1. Preliminary Obsevations on Large-Scale/Far-Field and Small-Scale/Near-Field Experiments

**Figure 2:**
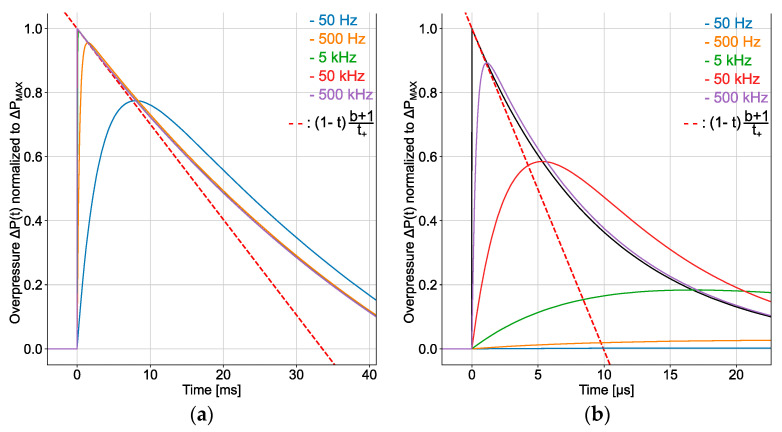
Friedlander waveform (in black) applied to the input of the first-order low-pass filter that models the frequency response of a pressure sensor for the side-on pressure measurement, and the waveform delivered at the output of the filter for various cut-off frequency $f_{c}$ (50 Hz in blue, 500 Hz in orange, 5 kHz in green, 50 kHz in red and 500 kHz in purple): (**a**) far-field and large-scale surface burst and side-on pressure measurement, and (**b**) small-scale and near-field surface burst and side-on pressure measurement. The ordinate is the ratio $\Delta P\left(t\right)/\Delta P_{MAX}$.

Consider two different Friedlander waveforms obtained respectively from large-scale $\left(m_{TNT}=1000\;kg\right)$ and far-field (Z=10 m/kg^1/3^) experiments, and a small-scale $\left(m_{TNT}=100\;g\right)$ and near-field (Z=0.3 m/kg^1/3^) experiment. Blast wave descriptors derived from K&B data are $\Delta P_{MAX}≅$ 15 kPa, $t_{+}≅$ 48 ms, and $I_{+}$ ≅ 310 kPa.ms (and b≅ 0.4) for the large-scale/far-field experiment, while $\Delta P_{MAX}≅$ 10 MPa, $t_{+}≅$ 100 µs, and $I_{+}≅$ 100 kPa.ms (and b≅ 9) for the small-scale/near-field experiment. These two Friedlander waveforms are applied to the input of first-order low-pass filters of different cut-off frequencies $f_{c}$ ranging from 50 Hz to 500 kHz. The waveforms at the filters output are displayed in Figure 2. It can be observed from Figure 2a that a sensor with the bandwidth of 50 kHz placed at the distance of 100 m from an explosive load with equivalent TNT mass of 1000 kg may be used to accurately estimate the magnitude of the overpressure peak. However, according to Figure 2b, this sensor provides a poor estimation accuracy of the peak magnitude when it is located at 15 cm from an explosive load with an equivalent TNT mass of 100 g. These simple examples illustrate that near field and small-scale experiments require a much larger sensor bandwidth than far field and large-scale experiments. In view of this result, it is not surprising that the magnitude estimation of overpressure peak in the near field and small-scale experiments reported in [3,4,6,15] is much less accurate than that obtained from the large scale and far field experiments presented in [7,19].

### 3.2. Response Time of a Pressure Sensor Dedicated to Blast Wave Experiments

When a Friedlander waveform is applied to the sensor input at time t=0, the voltage at the sensor output reaches its maximum value after some delay τ called the *response time* of the sensor. If the frequency bandwidth of the sensor had been infinite, then τ=0 and at time t=0, the voltage at the sensor output would have reached its maximum value $k_{s}\Delta P_{MAX}$, where $k_{s}$ is the sensitivity of the transducer. However, the bandwidth is finite in practice and, consequently, after some delay τ(≠0), the output voltage reaches its maximum, denoted by $\Delta V_{MAX}$, which differs from $k_{s}\Delta P_{MAX}$. Let us now determine the response time τ.

The Laplace transform v(s) of the output voltage is derived from Equation (2). We obtain:

(3) $$v\left(s\right)=k_{s}\frac{\omega_{c}}{s+\omega_{c}}\;p\left(s\right)$$

where the Laplace transform p(s) of the Friedlander waveform ΔP(t) of Equation (1) is given by:

(4) $$p\left(s\right)=\Delta P_{MAX}\frac{s+\frac{b-1}{t_{+}}}{{\left(s+\frac{b}{t_{+}}\right)}^{2}}$$

and from Equation (3), we derive:

(5) $$\;v\left(s\right)=k_{s}\Delta P_{MAX}\left[\frac{s+\frac{b-1}{t_{+}}}{{\left(s+\frac{b}{t_{+}}\right)}^{2}}+\frac{\left(A-1\right)s+B}{{\left(s+\frac{b}{t_{+}}\right)}^{2}}+\frac{C}{s+\omega_{c}}\right]$$

where

(6) $$A=\omega_{c}\;\frac{\omega_{c}-\frac{b-1}{t_{+}}}{{\left(\omega_{c}-\frac{b}{t_{+}}\right)}^{2}}=-C\;\mathrm{and}\;B={\left(\frac{b}{t_{+}}\right)}^{2}\;\frac{\omega_{c}-\frac{b-1}{t_{+}}}{{\left(\omega_{c}-\frac{b}{t_{+}}\right)}^{2}}$$

From the inverse Laplace transformation of Equation (5), the output voltage V(t) in the time-domain (for t>0) can be derived:

(7) $$\begin{array}{ll} V\left(t\right)=k_{s}\Delta P_{MAX} & \left(1-\frac{t}{t_{+}}\right)e^{-b\frac{t}{t_{+}}}\\ & +k_{s}\Delta P_{MAX}\left[\left(A-1\right)\left(1-b\frac{t}{t_{+}}\right)e^{-b\frac{t}{t_{+}}}+Bte^{-b\frac{t}{t_{+}}}+Ce^{-\omega_{c}t}\right] \end{array}$$

At time τ, the magnitude of the output voltage V(t) reaches its maximum value and, consequently, the time-derivative of this voltage cancels. The time τ is then the solution of the following equation:

(8) $$\begin{array}{ll} {\frac{dV}{dt}|}_{t=\tau }=0= & -k_{s}\Delta P_{MAX}\left[1+b\left(1-\frac{\tau }{t_{+}}\right)\right]\frac{e^{-b\frac{\tau }{t_{+}}}}{t_{+}}\\ & +k_{s}\Delta P_{MAX}[\left(1-A\right)\left(2-b\frac{\tau }{t_{+}}\right)\frac{b}{t_{+}}e^{-b\frac{\tau }{t_{+}}}+B\left(1-b\frac{\tau }{t_{+}}\right)e^{-b\frac{\tau }{t_{+}}}\\ & -\omega_{c}Ce^{-\omega_{c}\tau }] \end{array}$$

In order to derive a closed-form expression for the response time, we assume now that $b\tau \ll t_{+}$ and $\omega_{c}t_{+}\gg b$ (this assumption is often valid in practice but, for the sake of generality, it will not be applied in Section 3.6 and a numerical solution for the bandwidth will be derived). Therefore, A≅1, C≅−1, $\omega_{c}B≅{\left(\frac{b}{t_{+}}\right)}^{2}$ and Equation (8) can be simplified as follows:

(9) $$0≅-e^{-b\frac{\tau }{t_{+}}}\left(1+b+\frac{b^{2}}{t_{+}\omega_{c}}\right)+\omega_{c}t_{+}e^{-\omega_{c}\tau }$$

It follows that the response time τ of the sensor can be approximated from Equation (10):

(10) $$\tau ≅\frac{\ln r_{\omega \tau }}{\omega_{c}}\;\;\;\text{with}\;\;\;\;r_{\omega \tau }=\frac{\omega_{c}t_{+}}{1+b}$$

As $\omega_{c}t_{+}\gg b$, equation (10) is valid when $\tau_{\omega \tau }\gg 1$. As expected, τ→0 when $\omega_{c}\to \infty .$

### 3.3. Accuracy of the Overpressure Peak Estimation from Pressure Sensor in Blast Experiments

At time τ, the voltage V(τ) at the output of the sensor reaches its maximum value $\Delta V_{MAX}$ given by:

(11) $$\Delta V_{MAX}=k_{s}\Delta P\left(\tau \right)$$

As a matter of fact, we derive from Equation (3) that:

(12) $$sv\left(s\right)+\omega_{c}v\left(s\right)=\omega_{c}k_{s}p\left(s\right)\;$$

and since V(0)=0 at time t=0 (see Equation (7)), the inverse Lapace transformation of Equation (12) gives:

(13) $${\frac{dV}{dt}|}_{t=\tau }+\omega_{c}\Delta V_{MAX}=\omega_{c}k_{s}\Delta P\left(\tau \right)$$

from which Equation (11) can be derived, since $\;{\frac{dV}{dt}|}_{t=\tau }=0$ and $\omega_{c}\neq 0$. Consequently, according to Equations (1) and (11) we can estimate the voltage V(τ) as follows:

(14) $$\Delta V_{MAX}=k_{s}\Delta P_{MAX}e^{-b\frac{\tau }{t_{+}}}\left(1-\frac{\tau }{t_{+}}\right)≅k_{s}\Delta P_{MAX}\;\left[1-\left(b+1\right)\frac{\tau }{t_{+}}\right]$$

where the response time τ is estimated by Equation (10). As expected, the voltage $\Delta V_{MAX}$ differs from $k_{s}\Delta P_{MAX}$ for finite $\omega_{c}$ ($\Delta V_{MAX}\to k_{s}\Delta P_{MAX}$ as $\omega_{c}\to \infty )$. From the direct measurement of $\Delta V_{MAX}$ and the knowledge of the transducer sensitivity $k_{s}$, an estimation $\Delta {\hat{P}}_{MAX}$ of the overpressure peak can be obtained as follows:

(15) $$\Delta {\hat{P}}_{MAX}=\frac{\Delta V_{MAX}}{k_{s}}$$

From Equation (14), the estimation accuracy $k_{mes}\;$of the overpressure peak is then given by:

(16) $$k_{mes}=\frac{\Delta P_{MAX}-\Delta {\hat{P}}_{MAX}}{\Delta P_{MAX}}≅\left(b+1\right)\frac{\tau }{t_{+}}≅\frac{\ln r_{\omega \tau }}{r_{\omega \tau }}\;\;\mathrm{for}\;r_{\omega \tau }\gg 1$$

**Figure 3:**
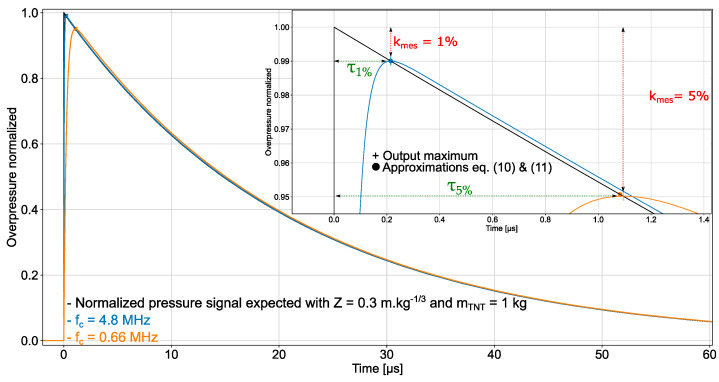
Friedlander waveform (derived from K&B data with Z = 0.3 m.kg^−1/3^ and $m_{TNT}$ = 1 kg, surface burst and side-on pressure measurement) applied to the input of two first-order low-pass filters with different cut-off frequency $f_{c}$ (4.8 MHz and 660 kHz) and waveforms delivered at the filters’ output. The ordinate is the ratio $\Delta P\left(t\right)/\Delta P_{MAX}$.

For the sake of illustration, Figure 3 displays the Friedlander waveform applied to the input of two first-order low-pass filters with different frequency bandwidths and the waveforms delivered at the filters output (note that $b\tau \ll t_{+}$ and $\omega_{c}t_{+}\gg b$ for $k_{mes}$ < 5% and consequently, Equation (10) is valid). It can be concluded that a very good estimation accuracy $k_{mes}$ of the ground truth overpressure peak $\Delta P_{MAX}$ can be expected from both sensor bandwidths, since $k_{mes}=$ 1% for $f_{c}=$ 4.8 MHz and $k_{mes}=$ 5% for $f_{c}=$ 660 kHz. Nowadays, oscilloscopes with the frequency bandwidth of 100 MHz can perform measurement with an accuracy of 1% [31]. Therefore, it is conceivable to design sensors that achieve the estimation accuracy of 1% on the overpressure peak magnitude.

### 3.4. Sensor Bandwidth Needed to Ensure a Desired Estimation Accuracy on the Overpressure Magnitude

**Figure 4:**
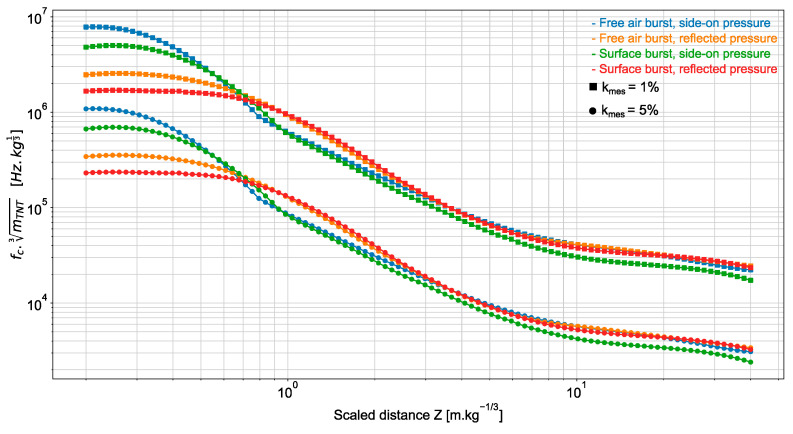
Scaled frequency bandwidth $\left(\sqrt[{3}]{m_{TNT}}f_{c}\right)$ as a function of the scaled distance Z for ensuring an estimation accuracy $k_{mes}\;$ of the overpressure peak magnitude of 1% (square marker) and 5% (point marker). The ground truth overpressure $\Delta P_{MAX}$ is obtained from K&B data.

From Equation (16), we can derive the sensor frequency bandwidth required to estimate the magnitude of the overpressure peak with the desired accuracy $k_{mes}$. Since the positive phase duration $t_{+}$ depends linearly on $\sqrt[{3}]{m_{TNT}}$ [1], the bandwidth varies as the inverse of $\sqrt[{3}]{m_{TNT}}$ at a given scaled distance and for a given estimation accuracy. For example, if $m_{TNT}$ is multiplied by 1000, $t_{+}$ is multiplied by 10, $f_{c}$ is divided by 10 and consequently $r_{\omega \tau }$ is unchanged. Figure 4 displays the frequency bandwidth of a sensor as a function of the scaled distance Z. This bandwidth guarantees an estimation accuracy $k_{mes}$ of 1% or 5% on the overpressure peak magnitude. In particular, for a sensor placed 0.5 m from an explosive load of 1 kg equivalent mass of TNT (as, e.g., in the blast experiment reported in [5]), the bandwidth of 3 MHz is sufficient to ensure the estimation accuracy of 1%, whereas only 30 kHz is needed if the sensor is placed at 30 m from the load.

### 3.5. Minimal Distance between the Sensor and Explosive Load to Ensure a Desired Estimation Accuracy on the Overpressure Peak Magnitude

In the example of Figure 2, we observe that a sensor with a frequency bandwidth of 50 kHz can be used to accurately estimate the overpressure peak $\Delta P_{MAX}$, if it is placed at 100 m from an explosive load of 1000 kg equivalent mass of TNT. However, this sensor fails to accurately estimate the peak magnitude when it is placed 15 cm from a load of 100 g equivalent mass of TNT. 

**Figure 5:**
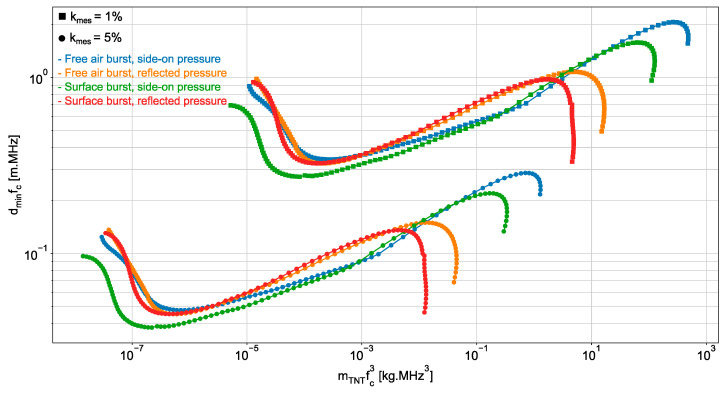
$d_{min}f_{c}\;$ as a function of $m_{TNT}f_{c}^{3}$ where $d_{min}$ denotes the minimal distance between the sensor and explosive load of equivalent TNT mass $m_{TNT}$ which is required to estimate the overpressure peak magnitude with the estimation accuracy $k_{mes}$ of 1% (square marker) or 5% (point marker).

To keep the estimation accuracy unchanged, the smaller the equivalent mass of TNT explosive of the source of the blast wave, the wider the sensor bandwidth. In very large-scale experiments, sensors with narrow bandwidth can be used to estimate the overpressure peak with high accuracy, while broadband sensors are needed in very small-scale experiments. In other words, when an explosive load is too small, pressure sensors cannot be used to estimate the magnitude of the overpressure peak with a high accuracy of 5% or less, whatever the distance between the sensors and load. Conversely, when the load is very large, sensors can be placed at any distances from the load to accurately estimate the peak. For intermediate explosive load of equivalent TNT mass $m_{TNT}$, there is a minimal distance $d_{min}\;$between the sensor and load to estimate the overpressure peak magnitude with the desired estimation accuracy $k_{mes}$. As a matter of fact, according to Equation (16), if an estimation accuracy $k_{mes}$ on the magnitude of the overpressure peak is required, then $r_{\omega \tau }=\omega_{c}t_{+}/\left(1+b\right)$ can be computed. As $t_{+}$ depends linearly on $\sqrt[{3}]{m_{TNT}}$ and, $t_{+}$ and b vary with the scaled distance $Z=R/\sqrt[{3}]{m_{TNT}}$ [20,21], the computed value of $r_{\omega \tau }$ allows the derivation of $\sqrt[{3}]{m_{TNT}}f_{c}$, or equivalently $m_{TNT}f_{c}^{3}$, from the scaled distance. Consequently, at the given distance $R=d_{min}$ where the estimation accuracy $k_{mes}$ must be achieved, $m_{TNT}f_{c}^{3}$ can be derived from the value of $d_{min}f_{c}$. For illustration purpose, $d_{min}f_{c}\;$is displayed in Figure 5 as a function of $m_{TNT}f_{c}^{3}$ for two estimation accuracies $k_{mes}$. We observe that, at the distance $d_{min}$ from a pressure sensor of fixed frequency bandwidth, if the estimation accuracy on the overpressure peak magnitude is 1% for an explosive load of equivalent mass $m_{TNT}$ of TNT, then the accuracy will be 5% for an equivalent TNT mass of $m_{TNT}/100$.

The variation in the pressure at the front of a blast wave generated by the explosion of $2\times 10^{6}$ kg of *Ammonium Nitrate Fuel Oil* (ANFO) is reported in [19]. If we assume that the bandwidth sensor (not specified in [19]) is of 100 kHz, we derive that $m_{TNT}f_{c}^{3}\;$ is of 2 × 10^3^ kg.MHz^3^ and, consequently, it can be concluded from Figure 5 that there is no minimal distance $d_{min}$ between the sensor and explosive load to estimate the overpressure peak magnitude with the estimation accuracy $k_{mes}$ up to 1%. The explosive load is large enough to place the sensor at any distances from the load and accurately estimate the magnitude of the pressure peak. If we assume that the same sensor (bandwidth of 100 kHz) is used in the experiment reported in [4], where the variation of the pressure at the front of a blast wave generated by the explosion of $m_{TNT}=$60 mg of equivalent mass of TNT is reported, then we obtain that $m_{TNT}f_{c}^{3}\;$ is of 6 × 10^−8^ kg.MHz^3^. If the accuracy of 1% is required in the magnitude estimation of the overpressure peak, there is again no minimal distance $d_{min}$ between the sensor and explosive load to estimate the overpressure peak magnitude with the estimation accuracy $k_{mes}$ of 1%. The equivalent mass of TNT explosive is too small for achieving the required accuracy, whatever the distance between the sensor and load. However, if the estimation accuracy of 5% is desired, then it can be concluded from Figure 5 that the load-to-sensor distance can be set to 1 m (in this case, the scaled distance would be of 26 m.kg−13).

### 3.6. Accuracy of the Overpressure Peak Estimation as a Function of the Sensor Bandwidth

The estimation accuracy $\;k_{mes}$ given in equation (16) is valid only for $r_{\omega \tau }\gg 1$. This restriction allows the derivation of a convenient closed-form expression for $k_{mes}$. We now compute $k_{mes}=\left(\Delta P_{MAX}-\Delta {\hat{P}}_{MAX}\right)/\Delta P_{MAX}$ as a function of the frequency bandwidth without formulating such restriction. 

The computation is organized as follows: (1) for each scaled distance Z and equivalent TNT mass $m_{TNT}$ of explosive loads, we compute the blast wave descriptors $\Delta P_{MAX}$, $I_{+}$ and $t_{+}$ from K&B data; (2) the resulting Friedlander waveforms are filtered by first-order low-pass filters with different cutoff frequencies ranging from $\left(b+1\right)/t_{+}$ to $1000\times \left(b+1\right)/t_{+}$; (3) the maximum voltage at each filter output is calculated and the estimation accuracy $\left(\Delta P_{MAX}-\Delta {\hat{P}}_{MAX}\right)/\Delta P_{MAX}$ is finally computed. The proposed method predicts the sensor bandwidth that allows estimating the overpressure magnitude at the front of a blast wave, in order to obtain a desired estimation accuracy $k_{mes}$.

**Figure 6:**
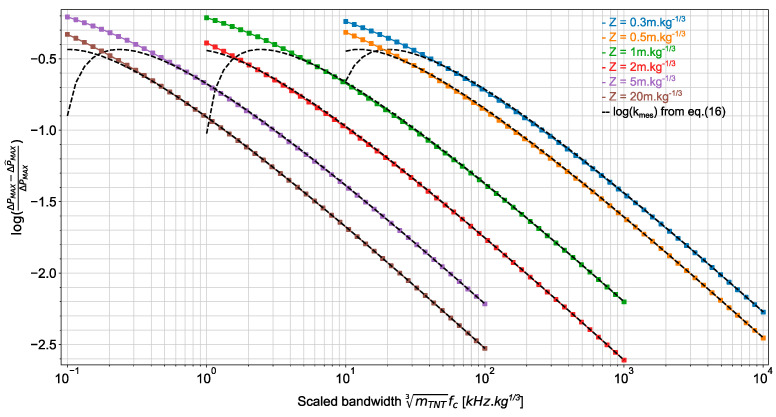
Estimation accuracy $\left(\Delta P_{MAX}-\Delta {\hat{P}}_{MAX}\right)/\Delta P_{MAX}$ on the overpressure peak as a function of the scaled bandwidth $\sqrt[{3}]{m_{TNT}}f_{c}$ for various scaled distances. Results are obtained for the side-on overpressure measurement and surface bursts.

As illustrated in Figure 6, the estimation accuracy of the side-on overpressure magnitude can be conveniently displayed as a function of the scaled bandwidth $\sqrt[{3}]{m_{TNT}}f_{c}$ for various scaled distances. It can be observed that the approximation provided by Equation (16) is in good agreement with the computed accuracy for $k_{mes}$ up to 10%.

## 4. Conclusions

In this paper, we have determined the frequency bandwidth of sensors required to estimate the overpressure magnitude at the front of a blast wave generated by an explosion, in order to obtain a desired estimation accuracy of this magnitude. The bandwidth depends on the scaled distance and equivalent mass of TNT of the explosive load. Friedlander waveforms were used to model the overpressure at the front of the blast wave. The descriptors of this wave were provided by Kingery and Bulmash data. In addition, the frequency response of pressure sensors was modelled by the transfer function of a first-order low-pass filter. A closed-form expression of the bandwidth has been proposed. 

Based on the analysis reported here, it can be concluded that it is not necessary to make a breakthrough to design ultra-wideband pressure sensors in order to accurately estimate the overpressure peak in the blast wave experiments reported in [19,32]. Available pressure sensors (see, e.g., [25]) can actually be used in such experiments. However, pressure sensors with a wider bandwidth of a tenth of MHz (see, e.g., [12,13]) are required in the experiment of [5] to estimate the overpressure peak with an accuracy $k_{mes}$ up to 1%. For very small explosive load experiments [4], we anticipate that a sensor bandwidth of a hundred MHz is needed to achieve an estimation accuracy $k_{mes}$ of 1%. 

As recently reported in [33], not only the pressure sensor but the whole measurement chain may impact the estimation accuracy on the magnitude of the overpressure peak during blast experiments. In particular, long coaxial cables between the pressure sensor and the acquisition unit may significantly impact the bandwidth of the system and, consequently, the measurement accuracy. Moreover, only the sensors have been considered in this work to derive the bandwidth requirement for estimating the overpressure magnitude at the front of a blast wave. However, the predicted estimation accuracy may not be obtained in practice, due to the impact of other complex factors involved in blast experiments (see, e.g., [10]). 

## Data Availability

The data presented in this study are available on request from the corresponding author.
